# Neuroprotective Potential and Paracrine Activity of Stromal Vs. Culture-Expanded hMSC Derived from Wharton Jelly under Co-Cultured with Hippocampal Organotypic Slices

**DOI:** 10.1007/s12035-017-0802-1

**Published:** 2017-11-13

**Authors:** Sylwia Dabrowska, Joanna Sypecka, Anna Jablonska, Lukasz Strojek, Miroslaw Wielgos, Krystyna Domanska-Janik, Anna Sarnowska

**Affiliations:** 1 Mossakowski Medical Research Centre, Polish Academy of Sciences, 5 Pawinskiego Street, Warsaw, Poland; 2 1st Department of Obstetrics and Gynecology, Medical University of Warsaw, Warsaw, Poland

**Keywords:** Wharton jelly, Stromal cells, Neuroprotection, Stem cells, Secretom, Hippocampal organotypic slice culture

## Abstract

Regardless of enormous translational progress in stem cell clinical application, our knowledge about biological determinants of transplantation-related protection is still limited. In addition to adequate selection of the cell source well dedicated to a specific disease and optimal standardization of all other pre-transplant procedures, we have decided to focus more attention to the impact of culture time and environment itself on molecular properties and regenerative capacity of cell cultured in vitro. The aim of this investigation was to determine neuroprotection-linked cell phenotypic and functional changes that could spontaneously take place when freshly isolated Wharton’s jelly mesenchymal stem cell (WJ-MSC) undergo standard selection, growth, and spontaneous differentiation throughout passaging in vitro. For determining their neuroprotective potential, we used experimental model of human WJ-MSC co-culture with intact or oxygen-glucose-deprived (OGD) rat organotypic hippocampal culture (OHC). It has been shown that putative molecular mechanisms mediating regenerative interactions between WJ-MSC and OHC slices relies mainly on mesenchymal cell paracrine activity. Interestingly, it has been also found that the strongest protective effect is exerted by the co-culture with freshly isolated umbilical cord tissue fragments and by the first cohort of human mesenchymal stem cells (hMSCs) migrating out of these fragments (passage 0). Culturing of WJ-derived hMSC in well-controlled standard conditions under air atmosphere up to fourth passage caused unexpected decline of neuroprotective cell effectiveness toward OGD-OHC in the co-culture model. This further correlated with substantial changes in the WJ-MSC phenotype, profile of their paracrine activities as well as with the recipient tissue reaction evaluated by changes in the rat-specific neuroprotection-linked gene expression.

**Electronic supplementary material:**

The online version of this article (10.1007/s12035-017-0802-1) contains supplementary material, which is available to authorized users.

## Introduction

Over the last few years, increasing number of investigations confirmed pro-regenerative, anti-inflammatory, and immunomodulatory potency of cellular therapy that can improve outcomes of brain injuries and block progression of some nowadays incurable neurodegenerative diseases together with reversing of their clinical symptoms. One of the most serious problems with the implementation of this therapy to clinic, however, is that a lot of undertaken trials show inhomogeneous and unstable efficiency partially due to differences in properties and regenerative capacity of the used human mesenchymal stem cell (hMSC) preparations.

Following available publications on performed clinical trials, one could often find inconsistency in preparation of delivered cells. The patients frequently receive freshly isolated stem cells as a first dose, whether for the next injections the cells are propagated by different periods of time in vitro*.* Despite that this prolonged culture could better fulfill increased needs for cell amounts to therapy, this would also change the cell properties important for their regenerative effectiveness.

Recently, the most commonly used cells in the regenerative medicine are human mesenchymal stem cells. hMSC can be derived from many different sources such as bone marrow, adipose tissue, umbilical or peripheral blood, and several other birthing tissues including umbilical cord [1, 2]. The last one isolated from umbilical cord stroma (Wharton jelly) have unique features making them preferable for CNS therapy. Their capabilities to differentiate toward non-mesenchymal cell lineages have been previously reported in several studies. In 2003, Mitchell et al. described differentiation of Wharton’s jelly mesenchymal stem cell (WJ-MSC) into the neurons in the conditioned medium [3]. In last few years, Messerliet al. [4] as well as our group have showed the intrinsic, spontaneous ability of WJ-MSC to differentiate toward neural cells [5, 6]. This neural differentiating subpopulation of WJ-MSC has been often named *pre*MSC [5], suggesting their derivation from very early developmental period [7]. Depending on defined culture conditions such as lowered oxygen tension and HIF alfa induction, WJ-MSC enhance these features being characteristic for embryonic-like, pluripotent stem cells (e.g., expression of pluripotency-associated genes like Oct-4, Nanog, and SOX-2) [6].

One of the existing hypotheses has assumed that these *pre*hMSC in adult stem cell niches could be direct successors of the neural crest stem cells, so as such well committed to triploblastic differentiation, expression of stemness-specific genes and ability to form the neurospheres in vitro [8]. By these properties, they are similar to embryonic stem cells but without their dangerous ability of tumor formation and with faster and longer self-renewal than all the rest of typical adult tissue-derived mesenchymal stem cells [9].

As already mentioned, final heterogenous WJ-tissue cell “product,” generally named as hMSC, still includes, besides the above stem/progenitors, also other lineage cells like endothelial, pericytes, and fibroblasts [10]. Any of these cell types can possess their own secretory properties and other interacting features. If their regenerative influence would be connected rather with a particular kind of stem/progenitor cells than with any other cell types, the best way to get optimal cellular product for tissue regeneration must correlate with their selection and purification. However, some authors claim that the therapeutically most efficient are naive, freshly isolated cells in spite of their cellular heterogeneity. There were some clinical trials in which fresh, untreated cells transplanted directly after isolation were found superior for treatment [11, 12]. In contrast, the cells from other, more selected passages and recommended as the neural-specific has occurred to be less efficient [13–15]. This is why it is essential to reconsider at which level of purification and differentiation mesenchymal stem cell preparations would be the most suitable for clinical application. In one of our previous studies, we evaluated how the neural commitment of the human umbilical cord blood progenitor cells (HUCB NSC) would influence therapeutic efficiency after systemic, intravenous delivery in the focal brain injury model in rats. The results showed that the best structural and functional improvement was achieved by transplantation of freshly isolated, non-selected HUCB mononuclear fraction [16].

Therefore, our recent study has been focused on the investigation of interrelation between the WJ-MSC phenotypic features (e.g., cell homogeneity and differentiation stages) and paracrine pro-regenerative and immunomodulatory cell properties. Moreover, we wanted to evaluate whether the neuroprotective capacity is stronger in freshly isolated stromal or differently timed cultured WJ-derived stem/progenitor cells. We hope that proper standardization of in vitro culture procedures could enhance the lifespan and increase the protective abilities of WJ-MSC for more efficient regenerative therapy of injured brain.

## Materials and Methods

### Isolation of Human Mesenchymal Stem Cells from Wharton’s Jelly

The umbilical cords were collected from the Obstetrics and Gynecology Clinic of the Infant Jesus Teaching Hospital in Warsaw. All parturient women agreed to use their afterbirth tissue in the experiments. The procedures of isolation were approved by the local ethical committee. The umbilical cords in sterile phosphate-buffered saline (PBS, Gibco) with penicillin/streptomycin (1:100, Gibco) were transported to the Neuro Repair Department of the Mossakowski Medical Research Centre Polish Academy of Sciences. Umbilical cords were washed multiple times with PBS containing penicillin/streptomycin in order to remove cord blood and then cut into 2-cm sections. Then Wharton’s jelly transplants (WJ) were isolated from the umbilical cord with dermatological punch 2 mm in diameter. WJ transplants were collected from the umbilical stroma located between vessels or near the periphery to omit contamination with pericytes. Isolated fragments were transposed to 25-cm^2^ polystyrene tissue culture flasks containing medium for hMSC culture (MSCGM™, Lonza) and incubated under air atmosphere at 37 °C and 5% CO_2_, with media replacement every 3 days. [17]. After 2 weeks, Wharton’s jelly fragments were removed and mesenchymal stem cells which adhered into the dishes were washed with PBS, trypsinized with 0.25% trypsin, and centrifuged in 1000 rpm for 3 min. Therefore, part of the isolated hMSC was seeded onto the polylysine cover glass slides in six-well plate in a density of 2 × 10^3^ cells/cm^3^. The rest of the cells were cultured to passage four and then seeded into the six-well plate in the same conditions. All mesenchymal stem cell cultures were performed in MSCGM™ medium at 37 °C, 21% O_2_, and 5% CO_2_.

### Organotypic Hippocampal Slice Culture

Organotypic hippocampal slices were prepared from 6- to 7-day-old Wistar rats derived from the Animal House of the Mossakowski Medical Research Centre. All experiments were approved by the local bioethical committee. Six- to seven-day-old rats were anesthetized and decapitated, and the brains were carefully removed and placed into the HBSS buffer (Gibco). After that, the hippocampi were isolated and cut into 400-μm slices using McIlwan tissue chopper. The healthy, presenting proper anatomy slices were chosen under the binocular and transposed onto the Millicell-CM membranes, with four hippocampal slices on each membrane. All procedures were performed on ice. Afterwards, the membranes with hippocampal slices were placed into the six-well dishes containing hippocampal slices medium with serum (50% Neurobasal (Gibco), 25% horse serum (Gibco), 22% HBSS (Gibco), 1 M HEPES (Sigma), glucose, and penicillin/streptomycin). Next day, the medium was changed and then replaced gradually by hippocampal slices medium free of serum (73% Neurobasal (Gibco), 22% HBSS (Gibco), 1 M HEPES (Sigma), B-27 supplements (Gibco), glucose, and penicillin/streptomycin). The organotypic hippocampal slices were incubated at 35 °C, 21% O_2_, and 5% CO_2_ for 7 days.

### Oxygen Glucose Deprivation

The oxygen glucose deprivation procedure was performed 7 days after hippocampal slice preparation and culture [18]. The Millicell-CM membranes with the organotypic cultures were transferred to six-well culture plates filled with 1 ml of Ringer solution, containing 10 mM Mannitol instead of glucose and saturated with a mixture of 95%N_2_/5%CO_2_. Plates were placed in a hypoxia chamber and exposed for 40 min at 37 °C to 95%N_2_/5%CO_2_ gas flow. After that time, the buffer was changed to serum-free medium and incubated in normoxic condition.

Quantification of cell death in the cornu ammonis (CA) region was performed as previously described [19]. The fluorescent cell death marker propidium iodide (PI) was added to the medium after 24 h of culture for 1 h. Fluorescent images were acquired using confocal laser scanning microscope LSM 510 (Zeiss). Damage was detected only in CA1 area, thus representing neuronal damage. To compare data from individual experiments, the mean densitometric value of the respective insult of an individual experiment (maximal fluorescent intensity) was set to 100% of maximal death values (MDVs). All other data were given in percent of this unprotected insult damage. Relative cell death was calculated from each standardized CA region as follows: % of dead cells = (experimental fluorescent intensity (FI) − background FI) / (maximal FI − background FI) × 100.

### The Organotypic Hippocampal Slices Co-Cultured in the Presence of Wharton’s Jelly Tissue Fragments or Wharton’s Jelly Mesenchymal Stem Cell Monolayer

The intact or oxygen-glucose-deprived (OGD) organotypic hippocampal slices were transposed to 6-well plates containing in lower chamber freshly isolated Wharton’s jelly fragments or mesenchymal stem cells and co-cultured in serum-free medium (73% Neurobasal (Gibco), 22% HBSS (Gibco), 1 M HEPES (Sigma), B-27 supplements (Gibco), glucose, and penicillin/streptomycin) at 35 °C, 21% O_2_, and 5% CO_2_ for 7 days (supplementary data). The culture of WJ fragments or WJ-MSC without hippocampal slices was performed as a control. The medium was changed every 2 days and collected at − 70 °C in order to further analyze the neurotrophic factor production.

In order to estimate the neuroprotective properties of Wharton’s jelly tissue fragments or mesenchymal stem cells, the organotypic hippocampal slice cultures after OGD were transferred to six-well dishes covered with WJ or WJ-MSC (80% confluence). Organotypic hippocampal culture (OHC) and cells were cultured in the serum-free medium (73% Neurobasal (Gibco) with 22% HBSS (Gibco), 1 M HEPES (Sigma), B-27 supplements (Gibco), glucose, and penicillin/streptomycin), but without direct cell-cell contact in the trans-well system for 24 h.

### Immunocytochemical Staining

Prior to the immunocytochemistry staining the cell cultures, freshly isolated or cultured WJ tissue fragments were washed with PBS, fixed with 4% paraformaldehyde for 15–30 min, and washed three times with PBS. Due to the volume of WJ fragments (1 mm × 1 mm), all the procedures were provided on orbital shaker with slow speed. The permeabilization was conducted with 0.2% Triton (Sigma) for 15 min and then unspecific epitopes were blocked by adding 10% goat serum (Gibco) for 1 h at RT. The primary antibodies diluted in PBS were used as listed in Table 1. All primary antibodies were transposed to the WJ or WJ-MSC cultures and incubated for 24 h at 4 °C. Next, the cell cultures or WJ tissue fragments were washed with PBS and the secondary antibodies diluted in PBS (1:1000) were applied: goat anti-mouse IgG1 for NF200, Ki67, nestin, and vimentin; goat anti-mouse IgM for A_2_B_5_ and NuMa; goat anti-mouse IgG2a for TUJ1; and goat anti-rabbit IgG(H + L) for fibronectin, GFAP, NG2, and GAP43. All secondary antibodies were conjugated with fluorescein isothiocyanate or Texas Red and incubated with WJ or WJ-MSC for 60 min at RT. Therefore, after washing three times with PBS, the nuclei were stained by 5 mM Hoechst 33258 (Sigma) for 15 min in RT. Afterward, the cell cultures or tissue fragments were washed with PBS; the glass slides were removed from the six-well dishes and cell cultures and were mounted with Fluoromount-G (Southern Biotechnology Association).

**Table 1:** Primary antibodies used for immunocytochemical staining

Primary antibodies	Isotype, dilutions	Company
Mouse monoclonal anti-Neurofilament 200 (NF200)	IgG1, 1:400	Sigma
Mouse monoclonal anti-A_2_B_5_	IgM, 1:300	Sigma/Millipore
Mouse monoclonal anti-nuclear mitotic apparatus protein (NuMa)	IgM, 1:50	Santa Cruz
Mouse monoclonal anti-neuron-specific class III beta-tubulin (TUJ1)	IgG2a, 1:500	Covance
Mouse monoclonal anti-Ki67	IgG1, 1:400	Novocastra
Mouse monoclonal anti-nestin	IgG1, 1:200	Millipore
Mouse monoclonal anti-vimentin	IgG1, 1:300	Dako
Rabbit polyclonal anti-fibronectin	IgG(H + L), 1:500	Dako
Rabbit polyclonal anti-glial fibrillary acidic protein (GFAP)	IgG(H + L), 1:200	Dako
Rabbit polyclonal anti-neuron-glial antigen 2 (NG2)	IgG(H + L),1:100	Millipore
Rabbit polyclonal anti-anti-growth associated protein-43 (GAP-43)	IgG(H + L), 1:500	Millipore

### Image Analysis and Statistic

Image analysis was performed using a Zeiss LSM 780 confocal laser-scanning microscope (Zeiss, Jena, Germany). Following acquisition, images were processed using Zeiss LSM software package v. 2.8 and Corel Draw v. 11.0. In order to analyze the number of cells positive for a particular antibody, the percent of immunoreactive cells was calculated from 200 counted Hoechst-positive (total) cells for each type of culture.

Statistical analysis of the data was conducted by one-way ANOVA followed by Bonferroni’s multiple comparison test. The values were considered as significant when *p* < 0.05. Data were presented as mean ± SE. The number of different experiment (*n*) and the number of slices used per group and per experiment are indicated for each experiment.

### Enzyme-Linked Immunosorbent Assay

Enzyme-linked immunosorbent assay was used to quantitate human cytokines and neurotrophic factors released by Wharton’s jelly fragments or dispersed mesenchymal stem cells cultured alone or co-cultured either with intact or OGD-injured hippocampal slices. All collected media were condensed by centrifuging in 4000 rpm for 20 min in the special test tubes Corning®Spin-X®UF Concentrator. The human colorimetric kit of ELISA Strip for Profiling 8 Cytokines (Signosis) containing interleukin 2 (IL-2), interleukin 6 (IL-6), interleukin 10 (IL-10), vascular-endothelial growth factor (VEGF), beta polypeptide nerve growth factor (β-NGF), basic fibroblast growth factor (FGFb), transforming growth factor β (TGF-β), and insulin-like growth factor 1 (IGF-1) was used. All the experiments were performed according to the manufacturer recommendations. The final concentration of cytokines and neurotrophic factors was estimated according to the determined standard curve of absorbance.

### Quantitative Real-Time Polymerase Chain Reaction Analysis of Rat Neurotrophic Factors

To detect the mRNA expression level of selected cytokines and growth factors, the RT-PCR method was used. Before the RT-PCR procedure, the total RNA from all experimental paradigms was isolated. The hippocampal slices were transposed from Millicell-CM membranes to TRIzol (Invitrogen) reagent and incubated for 5 min in RT. Then, chloroform was added for 3 min in RT, centrifuged for 15 min in 12,000 rpm at 4 °C, and the water phase was collected. After that, the samples were incubated with isopropanol for 10 min at room temperature, centrifuged for 10 min in 12,000 rpm at 4 °C, and the supernatant was ejected and 75% ethanol was added to the pellet. The solution was centrifuged for 5 min in 7500 rpm at 4 °C, the supernatant was ejected, and the pellet was dried, then re-suspended with RNA-free. The concentration and purity of isolated RNA was measured by the NanoDrop ND-1000 spectrophotometer, and the ratio from 260/280 and 260/260 was determined. Only non-contaminated samples with ratio > 1.8 were used for further procedures. RNA (1 μg) was used for cDNA preparation with use of the High-Capacity RNA-to-cDNA Kit (Applied Biosystem).

For quantitative real-time PCR (qRT-PCR), the 7500 Real-Time PCR thermocycler (Applied Biosystem) was used. The final probes contained cDNA, SYBR Green PCR Master Mix (Applied Biosystems), and gene-specific primers listed below (Table 2). Glyceraldehyde-3-phosphate dehydrogenase (GAPDH) was used as an internal control. RT-PCR was performed at 95 °C for 15 s and at 60 °C for 60 s in 40 cycles with initial activation of enzyme at 95 °C for 10 min. The normalized fold expression was obtained using the 2^−ΔΔCt^ algorithm. The results were presented as the normalized fold expression for each gene and compared to expression in OHC slices treated as a control.

**Table 2:** The primers used for qRT-PCR

Gene	Product size (bp)	Primer sequence (5′- > 3′)
EGF	74	Forward: TCTGGGCTCAGGACGGTAGA Reverse: ATGGATGGAGCCACCGTTATAG
VEGF	69	Forward: GAGGAAAGGGAAAGGGTCAAAA Reverse: CACAGTGAACGCTCCAGGATT
NT3	74	Forward: AGAGGCCACCAGGTCAGAATT Reverse: TGTAGCGTCTCTGTTGCCGTAGT
TGFβ	76	Forward: CGTGGAAATCAATGGGATCAG Reverse: CAGGAAGGGTCGGTTCATGT
BDNF	136	Forward: CGGCTGGTGCAGGAAAGCAA Reverse: TCAGGTCACACCTGGGGCTG
GDNF	144	Forward: AAGGTCGCAGAGGCCAGAGG Reverse: TCTCGGCCGCTTCACAGGAA
CNTF	78	Forward: GGTTATTCTGGCTATGCAAATGTG Reverse: GATAGGTGGGCCATCCATTTATT
IGF-1	145	Forward: CAGCATTCGGAGGGCACCAC Reverse: CATGTCAGTGTGGCGCTGGG
FGF2	166	Forward: ATCCCTCCCCAGTTCAGTTC Reverse: GCCTCCAAGTTGCAAAAAGA
HGF	101	Forward: CAATTTGGACCATCCTGTAATATCC Reverse: TCAAACTAACCATCCACCCTACTG
Bax	183	Forward: AACATGGAGCTGCAGAGGAT Reverse: GATCAGCTCGGGCACTTTAG
Bcl2	115	Forward: CTGGTGGACAACATCGCTCT Reverse: GCATGCTGGGGCCATATAGT
CASP3	159	Forward: GGACCTGTGGACCTGAAAAA Reverse: GCATGCCATATCATCGTCAG
Neuroglobin	161	Forward: AGCTTTAAAGGGCGGTTCTC Reverse: CAGGCCCAGTGCCTTAGTC
GAPDH	145	Forward: AGGGTGGTGGACCTCATGGC Reverse: AGTGCTCAGTGTTGGGGGCT

## Results

### Wharton’s Jelly Fragments and Wharton’s Jelly Mesenchymal Stem Cell Co-Culture Have Neuroprotective Effect on Hippocampal Slices

**Fig. 1:**
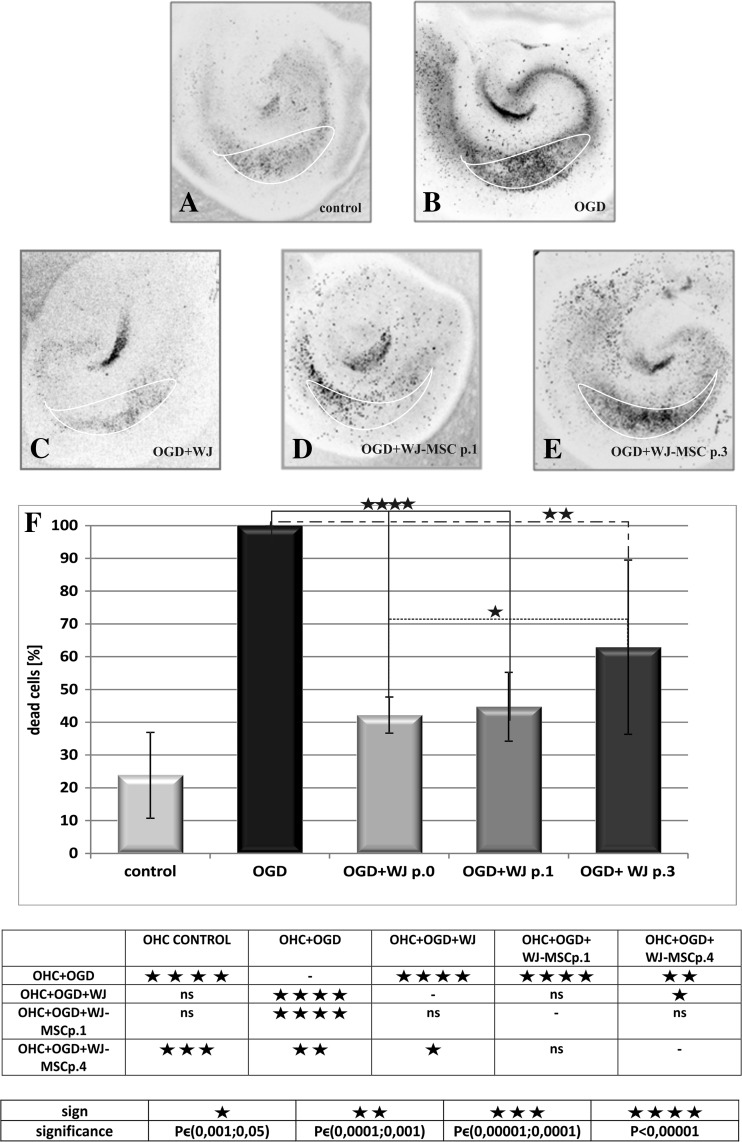
The analysis of neuroprotective properties of Wharton’s jelly mesenchymal stem cells depending on either the age of the culture and the degree of cell differentiation. The cell death was observed in hippocampal slices undamaged (**a**) and significantly increased in hippocampal slices damaged by glucose oxygen deprivation (**b**). The degree of cell death decreased after 24 h of co-culture with Wharton’s jelly fragments (**c**), freshly isolated mesenchymal stem cells passage 1 (**d**), and older Wharton’s jelly mesenchymal stem cell passage 3 (**e**). The diagram shows comparison between percent of cell death in total cells in hippocampal slices (**f**). The strongest neuroprotective properties belonged to WJ fragments (42.2%); WJ-MSC passage 1 had also strong ability for neuroprotection (44.7%), and weaker neuroprotective effect was observed after co-culture with WJ-MSC passage 4 (62.9%)

The neuroprotective potential of Wharton’s jelly fragments and Wharton’s jelly-derived mesenchymal stem cell monolayers was estimated in the model of indirect co-culture with organotypic hippocampal slices transiently deprived by oxygen and glucose (OGD) in vitro (Fig. 1). The neuroprotective effect of the cells at post-ischemic recovery has been measured as the decreasing number of cell death in the vulnerable CA1 region of hippocampal slices (OHC) labeled directly by the propidium iodide (PI).

According to the method elaborated in our laboratory, about 60% of the initial number of cells in the CA1 region of hippocampus died under employment of OGD injury [20]. In the presented results, the total number of dead cells counted in every unprotected OGD experiment in the CA1 region has been taken as 100% of the normalized maximal death value (MDV; Fig. 1b). In the naive slices incubated in standard culture conditions, the spontaneous cell death achieved 23.8 ± 13.1% of MDV (Fig. 1a). Co-cultures of OGD slices with Wharton’s jelly fragments significantly decreased the MDV value from the initial 100% in unprotected OGD slices down to 42.2 ± 5.5% (*P* < 0.001; Fig. 1c). Similarly, co-culture with WJ-MSC derived from the first passage in vitro (WJ-MSC p.1) decreased the cell death incidents in the CA1 region of OHC slices to 44.7 ± 10.5% (*P* < 0.001; Fig. 1d). However, as revealed by co-cultures of OGD hippocampal slices with the older WJ-MSC collected from the third or fourth passage (WJ-MSC p.3 or 4), the protective potency against OGD-induced OHC damage decreased to 62.9 ± 26.6% of MDV (*P* < 0.01%; Fig. 1e). Therefore, it seems that neuroprotective properties of WJ-MSC cultures diminished considerably along a time of cells grown in vitro (Fig. 1f).

### Wharton’s Jelly Fragments and Wharton’s Jelly Mesenchymal Stem Cell Monolayer Cultures Have a Spontaneous Ability to Neural Lineage Differentiation Further Enhanced by Co-Culturing with Oxygen-Glucose-Deprived Hippocampal Slices In Vitro

As described in the “Methods” section, in order to achieve a better understanding of possible mechanism(s) of neuroprotection exerted by WJ fragments or WJ-MSC, in the next experiments, a whole pallet of immunostaining was performed.

**Fig. 2:**
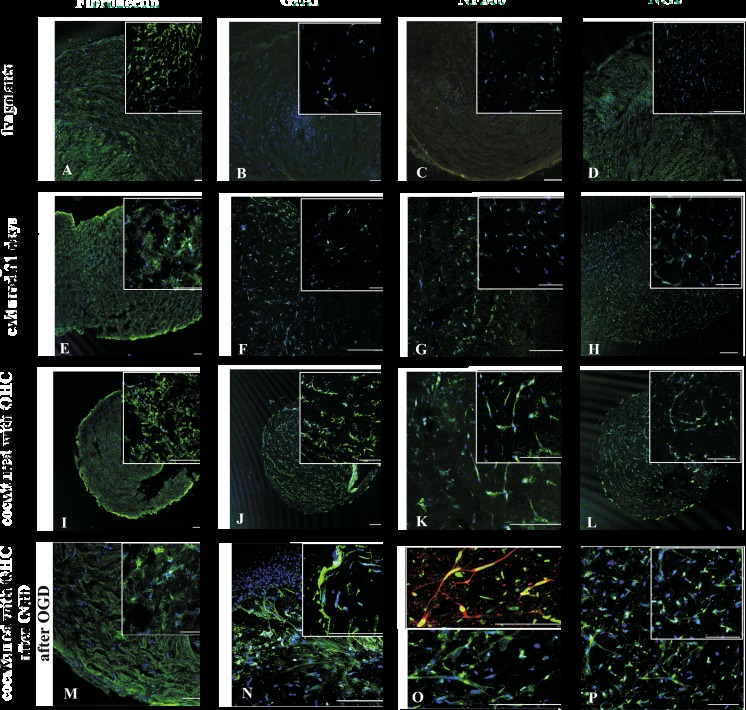
The immunocytochemical analysis of mesenchymal stem cells and neural cells markers expressed by WJ fragments in control conditions; they co-cultured with untreated hippocampal slices and with hippocampal slices damaged by oxygen glucose deprivation. The nuclei were stained with Hoechst (**m**–**p**) or NuMa (**o** upper picture). Our results show that mesenchymal stem cell marker fibronectin (**a**) was expressed more extensive in the naive culture than in those after 21 days of cultivation (**e**) or co-culturing with hippocampal slices injured or not (**i**, **m**) by OGD. Moreover, the expression of neuronal marker NF200 (**c**, **g**, **k**, **o** bottom), oligodendrocyte marker NG2 (D,H, L) and astrocyte marker GFAP (**b**, **f**, **j**, **n**), appeared or increased after co-culture with OHC and further potentiated when OHCs had been damaged by transient OGD episode. Scale bars of 100 μm are inserted on the bottom of pictures

The cells is freshly dissected or 7 div cultured WJ fragments positively and homogeneously immunoreacted to typical hMSC-specific antibody against fibronectin. In opposite, the WJ fragments co-cultured with the hippocampal slices presented evidently decrease in expression of this marker (Fig. 2a, e, i). Concomitantly, these freshly isolated as well as 7 div cultured WJ fragments expressed only minimal levels of the neural lineage-specific protein markers like astrocytic GFAP, neuronal NF200, and oligodendrocyte marker NG2 (Fig. 2b–d, f–h). However, after 7 days of their co-cultures with OHC slices, the expression of all these neuronal, astrocyte, and oligodendrocyte markers increased substantially (Fig. 2j–l) and has been even further potentiated when co-cultured slices were previously damaged by OGD treatments (Fig. 2m–p). In effect, the cells in co-cultured WJ fragments differentiated faster and displayed high expression of neural markers: GAP43, NF200, and GFAP.

**Fig. 3:**
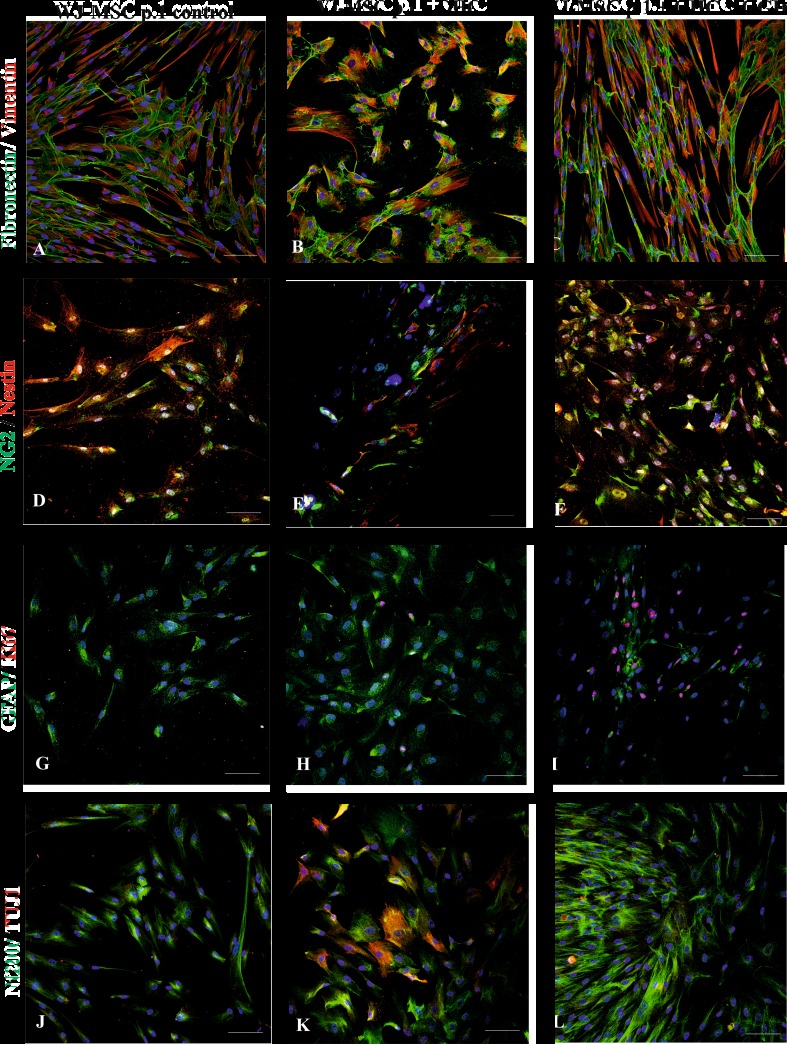
The immunocytochemical analysis of mesenchymal stem cell and neural cell markers expressed by WJ-MSC passage 1 in control conditions, co-cultured with untreated hippocampal slices and hippocampal slices damaged by oxygen glucose deprivation. The nuclei were stained with Hoechst. Our results shows that mesenchymal stem cell markers fibronectin (green) and vimentin (red) (**a**–**c**) were expressed similarly in different culture conditions. Moreover, the expression neuronal markers nestin (red) (**d**–**e**), Nf200 (green), TUJ1 (red) (**j**–**l**), oligodendrocyte marker NG2 (green) (**d**–**f**), and astrocyte marker GFAP (green) (**g**–**i**) increased after co-culturing with OHC and was the highest after co-culturing with OHC damaged by OGD. Therefore, the marker of proliferation Ki67 (red) was not observed in control culture (**g**), whereas it appeared after cultivation with untreated hippocampal slices (**h**) and further increased after co-culture with damage hippocampal slices (**i**). Scale bar 100 μm

Further, the effect of OHC slices co-cultured on the WJ-MSC derived from different passages has been tested. Co-culturing hippocampal slices with WJ-MSC derived from passage 1 caused mesenchymal markers (Fibronectin and Vimentin) to be co-expressed evenly in almost all of the cells (Fig. 3a–c) and did not change significantly after co-culture with injured OGD slices. In contrast, the neural progenitors/oligodendrocyte NG2 marker expressed in 17% of the whole number of WJ-MSC control cultures increased significantly to 28.5% after co-culturing them with control or damaged hippocampal slices (*P* < 0.01). The Nestin-specific staining was found positive in 13% of cells in control monolayer alone and elevated sharply after OHC co-cultured either control or OGD to 30 and 34%, respectively (*P <* 0.001). In parallel, the neuronal marker NF200 increased from 22.5% in control up to 34% (*P <* 0.01) upon co-culture with uninjured OHC and to 36.5% (*P <* 0.001) with injured slices. The more mature neuron marker TUJ1, being totally absent in control WJ-MSC culture at passage 1, started being expressed in 0.5% of cells co-cultured with uninjured OHC (*P <* 0.001) then increased to 6% in the presence of OGD-injured slices (*P* < 0.05). Interestingly, the astrocyte-specific protein (GFAP) stayed almost unchanged on the level of about 14% cells in all three types of tested experimental settings. The expression of cell proliferation marker Ki67 was absent in control culture, then slightly increased to 7.5% *(P <* 0.01) in co-culture with control OHC and even further increased to 12.5% (*P <* 0.0001) after co-culture with OGD-injured hippocampal slices (Fig. 5a).

**Fig. 4:**
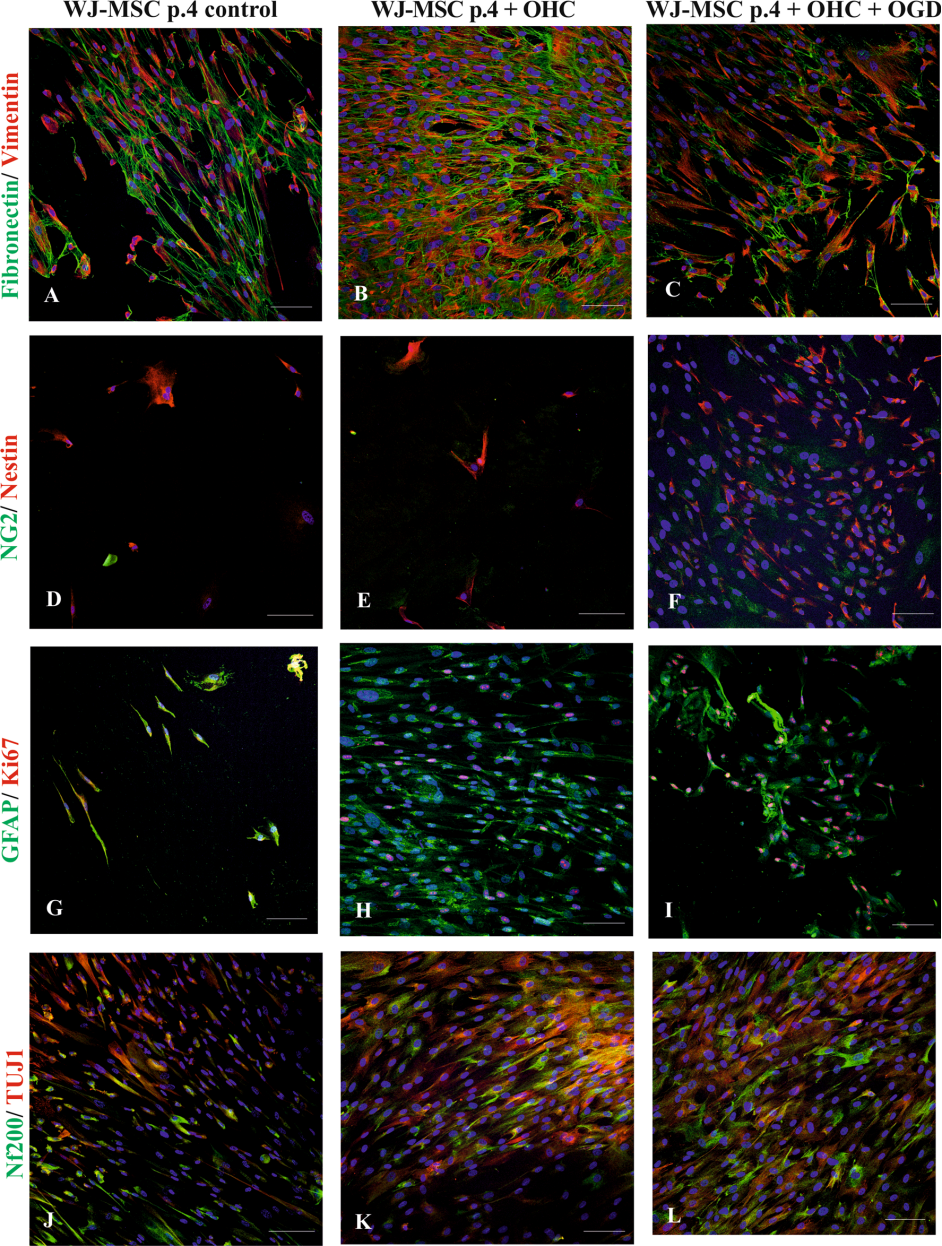
The immunocytochemical analysis of mesenchymal stem cell and neural cell markers expressed by WJ-MSC passage 4 in control conditions, co-cultured with untreated hippocampal slices and hippocampal slices damaged by oxygen glucose deprivation. The nuclei were stained with Hoechst. Our results shows that mesenchymal stem cells markers fibronectin (green) and vimentin (red) (**a**–**c**) were expressed similarly in different culture conditions. Moreover, the expression neuronal markers nestin (red) (**d**–**e**), Nf200 (green), TUJ1 (red) (**j**–**l**), oligodendrocyte marker NG2 (green) (**d**–**f**), and astrocyte marker GFAP (green) (**g**–**i**) increased after co-culturing with OHC and was the highest after co-culturing with OHC damaged by OGD. Therefore, the marker of proliferation Ki67 (red) was not observed in control culture (**g**), whereas it appeared after cultivation with untreated hippocampal slices (**h**) and further increased after co-culture with damage hippocampal slices (**i**). Scale bar 100 μm

Similar to WJ-MSC p.1, the WJ-MSC p.4 displayed a co-expression of fibronectin and vimentin (Fig. 4c). Interestingly, the percentage of positive cells in total cell population did not change significantly under different investigated co-culture settings as demonstrated precisely on the included diagram (Fig. 3c).

In WJ-MSC p.4, the number of neural progenitor markers also increased significantly in the presence of hippocampal slices and the increase was greater in cells co-cultured with OGD injured slices. Namely, the oligodendrocyte marker NG2 increased from 13.5% in control WJ-MSC to 27% after their co-culture with the intact OHC and to 29% after co-culture with the OGD-damaged OHC (*P <* 0.001). The expression of Nestin was detected in 24.5% of control cells, and this percentage significantly increased in the presence of both, uninjured (32%) and OGD injured (33.5%) hippocampal slices (*P <* 0.05). Similarly, immunostaining for another neuronal marker, NF200 increased from control 25.5 to 39% or 40% when WJ-MSC were cultured accompanied by the OGD-treated or untreated OHC slices respectively with *P* < 0.0001. Moreover, TUJ1 was detected in 17% at control cells and this number significantly increased to about 25% after co-culturing with uninjured as well as OGD-injured OHC slices (*P <* 0.01).

**Fig. 5:**
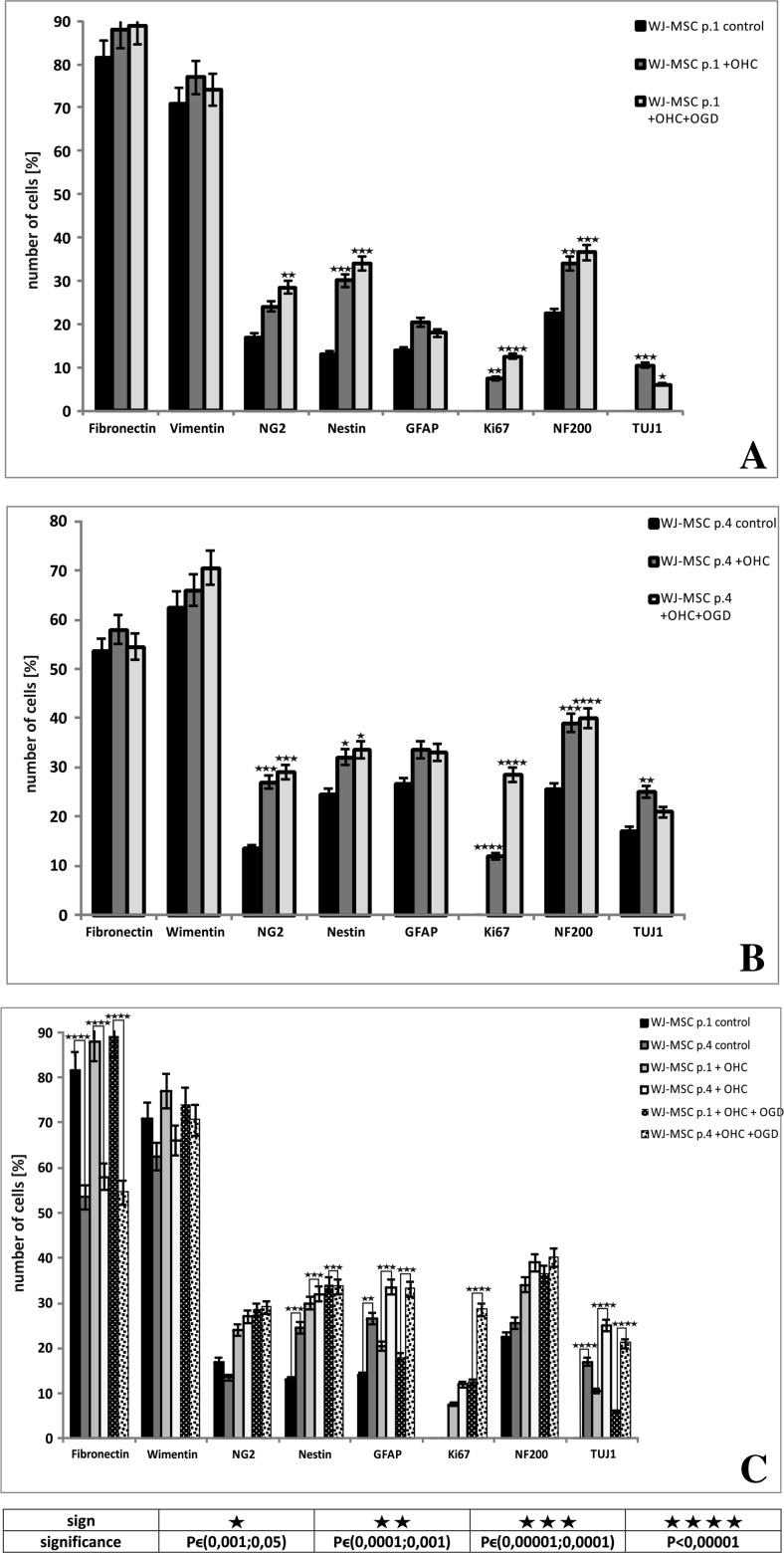
The quantitative immunocytochemistry analysis of number of Wharton’s jelly mesenchymal stem cells from passage 1 (**a**) and passage 4 (**b**) cultured in different cultivation conditions and comparison between number of markers expressed by cells from passages 1 and 4 (**c**). In both cases, mesenchymal stem cells markers fibronectin and vimentin were expressed in similar amount by each type of culture. The expression of neural markers NG2, Nestin, GFAP, NF200, TUJ1, and proliferation marker Ki67 increased after co-culturing with OHC and further increased after co-culturing with OHC damaged after OGD. The comparison showed that WJ-MSC p.1 expressed more mesenchymal stem cells markers, whereas WJ-MSC p.4 immunodetected more neural and proliferation markers in all types of the culture

Furthermore, WJ-MSC p.4 co-cultured with both types of hippocampal slices (injured or not) showed relatively stable level of astrocyte marker GFAP at 26.5% of cell population. Similarly to WJ-MSC p.1, the proliferation marker Ki67 was rarely or never expressed in the control WJ-MSC at p.4. Co-culture of these cells with control hippocampal slices increased the number of Ki67-positive cells to 12%, and the presence of OGD-injured OHC increased it even further to 28.5% (*P <* 0.0001) (Fig. 5b).

Comparing WJ-MSC cell passage 1 to that in passage 4 after 7 days of the above experimental co-cultures, we can state that mesenchymal stem cell marker fibronectin was expressed in significantly higher level by cells at p.1 (*P <* 0.0001). These p.1 cells displayed also morphology more typical for mesenchymal stem cells and were less differentiated toward neural lineages. However, the expression of the second mesenchymal marker—vimentin—did not differ significantly between the cells from the two tested passages. Moreover, comparing with passage 1, the WJ-MSC at p.4 presented significantly more neuronal markers: nestin (*P <* 0.001) and TUJ1 (*P <* 0.0001). Also, the expression of astrocyte marker GFAP was significantly higher in WJ-MSC p.4, either in control culture (*P <* 0.01), as after co-culture with OHC (*P <* 0.001) in comparison to WJ-MSC p1.. Additionally, the comparison showed significantly higher level of the proliferation marker Ki67 in WJ-MSC derived from passage 4 than from passage 1 in co-culture with OHC after OGD treatment (*P <* 0.0001) (Fig. 5c). Thus, WJ-MSC in p.4 displayed more mature but still proliferating phenotypes with ability to neural differentiation.

### Secretion of Human-Specific Neurotrophic Factors by Wharton’s Jelly Fragments and Wharton’s Jelly Mesenchymal Stem Cell Monolayer Cultures Is Significantly Modulated by the Presence of Rat Organotypic Hippocampal Culture Slices

Looking further on the molecular basis of interactions between potentially protective WJ-derived components (WJ tissue fragments or originating from them WJ-MSC cultures), and organotypic hippocampal slices in the OGD injury model, we measured release of several characteristic human proteins secreted during tissue repair processes.

The enzyme-linked immunosorbent assay (ELISA) indicated that specific expression of human neurotrophic factors changed in a manner dependent on the type and time of interactions between co-cultured cells and recipient effector tissue. The level of hVEGF secreted by WJ-MSC p.1 cells did not react to the presence of either intact or OGD-injured hippocampal slices. The significant increase in the expression of hVEGF was observed only in co-cultures of WJ fragments with OGD-injured OHC slices (*P* < 0.0001). In contrast to p.1, the secretion of hVEGF by WJ-MSC at p.4 was significantly elevated by both, intact and OGD injured OHC co-cultures (*P* < 0.0001).

**Fig. 6:**
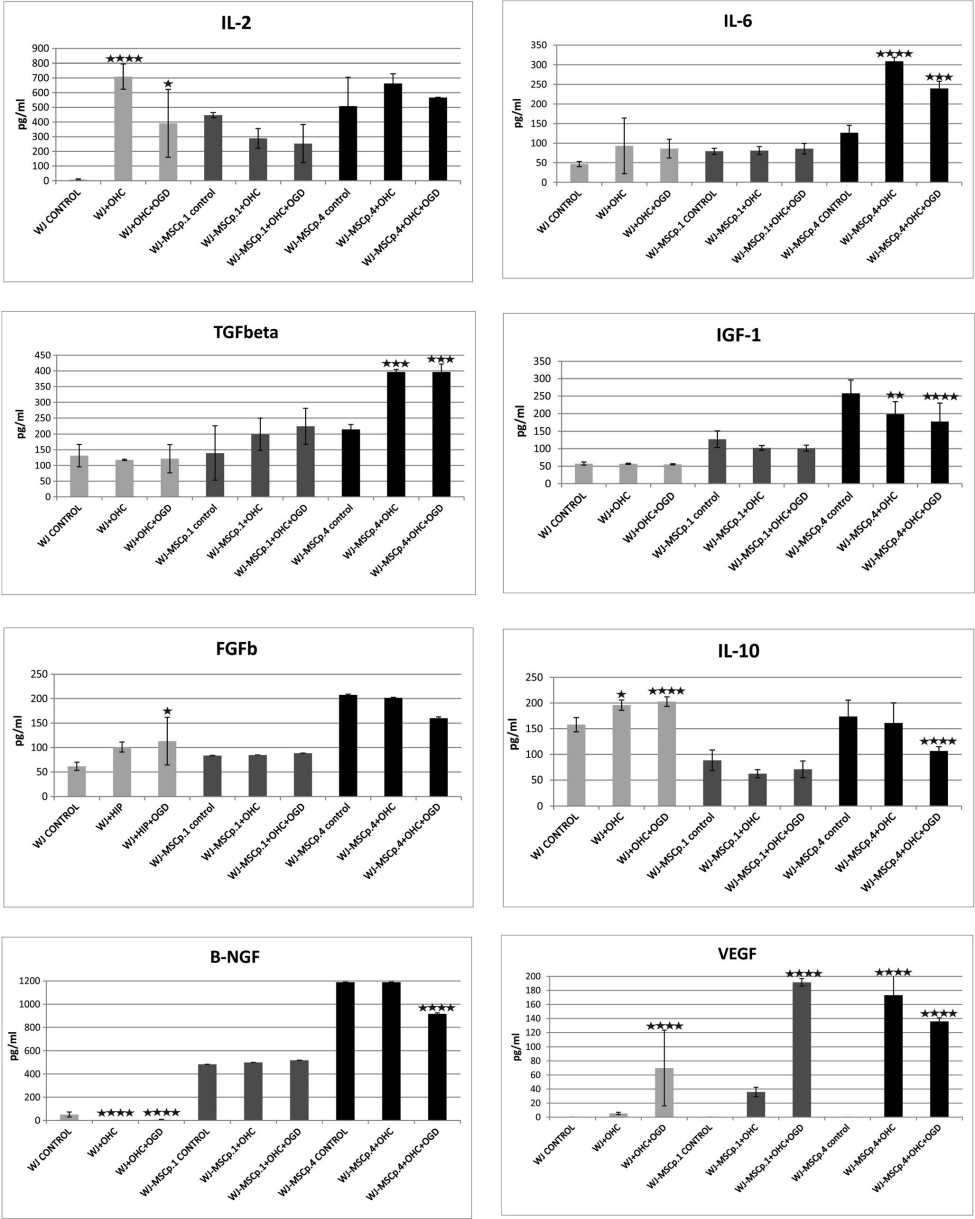
The enzyme-linked immunosorbent assay (ELISA) analysis of human neurotrophic factors and interleukins expressed by Wharton’s jelly fragments (WJ) and Wharton’s jelly mesenchymal stem cells (WJ-MSCs) derived from passages 1 and 4 cultured alone, co-cultured with intact rat hippocampal slices (OHC), and co-cultured with OHC damaged by oxygen glucose deprivation (OGD)

The analysis of IL-6 release profile did not exhibit any significant differences between secretion by WJ fragments or WJ-MSC p.1 co-cultures and without influence of pathophysiological status of the co-cultured OHC slices (see Fig. 1 and the reactions on WJ fragments in control culture, co-culture with OHC, and co-culture of the MSC passage-1 with the OHC after OGD in Fig. 6). Exclusively in the case of an older, 4th passage WJ-MSC reacted with increased release of IL-6 but only under co-culture with intact as well as injured hippocampal slices (*P* < 0.0001).

In the next experiment, we have demonstrated that initial levels of hβ-NGF is decreased significantly to almost undetectable amounts after co-culture of Wharton’s jelly fragments with either control or OGD-injured OHCs (*P* < 0.0001). Slight but still significant down-regulation of hβ-NGF secretion has been noticed in WJ-MSC- p.4 co-cultures but only in the presence of OGD injured OHC slices. For importance, we observed here general elevation of secreted hβ-NGF levels in all experiments performed on WJ-MSC p.4 cultures.

The enhanced secretion was also noticed for almost all examined factors released from the cells of WJ-MSC p.4 as compared to any other experimental settings. This was observed for TGFb, IL-2, IL-10, FGFb, and IGF1. What should be stressed here is that also WJ fragments exerted higher ability to secrete certain cytokines like IL-2, IL-10, and VEGF either spontaneously or under influence of hippocampal co-cultures in comparison to WJ-MSC p.1 (Fig. 6). Moreover, the only factor in WJ-MSC-p.1, whose release was markedly stimulated but only in the co-culture with OGD-injured hippocampal slices, was VEGF.

### The Changes of Neurotrophic Factors Genes Expression in Co-Cultured Rat Organotypic Hippocampal Slices in Response to the Different Experimental Arrangements

**Fig. 7:**
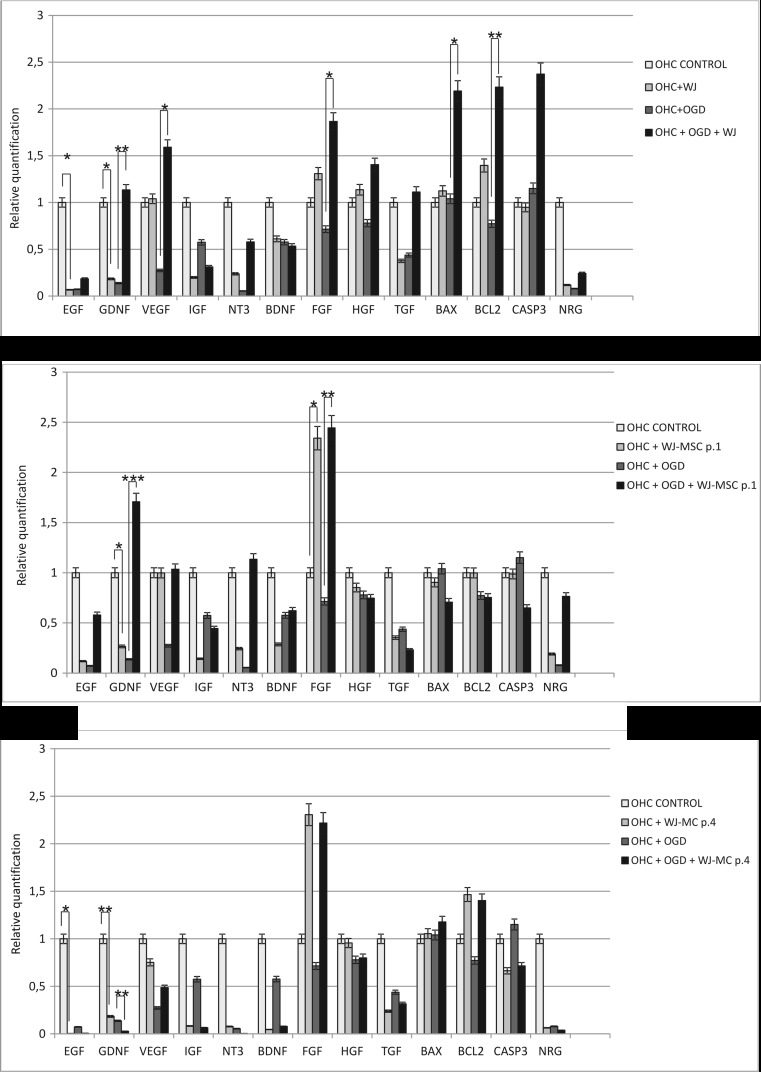
Relative quantification of rat neurotrophic factors expression in hippocampal slices intact or damaged by oxygen glucose deprivation cultured alone or co-cultured with Wharton’s jelly fragments (WJ) or Wharton’s jelly mesenchymal stem cells (WJ-MSCs) derived from passages 1 and 4; *n* = 6

In further experiments, we have explored transcriptional activation of several neuroprotection-linked genes in the OHC model under influence of the OGD injury and subsequent protection by their co-cultures with WJ tissue fragments or monolayer cultures. Our studies revealed that the expression of the majority of genes decreased in slices after oxygen glucose deprivation, with exception of the cell death-linked Bax and Casp3 which were activated by OGD. The real-time analysis showed that the neuroprotection effect of freshly isolated WJ fragments on injured slices could be correlated with the significantly increased expression of the EGF, GDNF, VEGF, FGF, Bax, and Bcl2. Furthermore, co-culture of OHC/OGD with WJ-MSC-p.1 also led to significant activation of neuroprotection-linked genes expression like GDNF and FGF in hippocampal slices (Fig. 7).

## Discussion

Progress in regenerative therapy is inseparably connected with further exploring of stem cell biology, their developmental lineages, differentiation, and distinguished features. Preclinical studies and first clinical trials have led to the conclusion that the currently running cell therapy is based mostly on the secretory properties of mesenchymal cells. Therefore, the final results of stem cell-based treatment may depend on many factors affecting secretive abilities of cells like proper selection of the optimal state of cell differentiation, choice between heterogenic, stromal cell population, e.g. SVF, and more homogenous stem/progenitor differentiating sub-populations, etc.

In this study, we highlighted significant differences in phenotype, secretory abilities, and progress in maturation level of WJ-MSC cultured in vitro for relatively short time up to four passages often considered as an early, optimal for treatment period. It has been shown that even for this minimal time, adherent culture could subject substantial changes in gene expression, differentiation, and secretory cell properties [21].

Our results, in accordance with other data, have confirmed that 3D tissue structure well retained in the umbilical cord fragments may inhibit WJ-MSC differentiation and keeps cells longer in their stemness stage. The earliest expression of neural-specific proteins appeared up only in the first passage, when cells migrated out from the 3D spatial structure to form an adherent 2D monolayer. Convergent results have been reported by Ropper group which in order to delay hMSC differentiation settled the cells into poly(lactic-co-glycolic) acid 3D scaffolds. The applied scaffolds maintained hMSC stemness and enhanced their positive effects on neighboring cells/tissue by enhancing their neurotropism, angiogenesis, neurogenesis, and anti-inflammatory functions [22]. However, the culture transition from 3D to 2D spatial structure is not a main or obligatory determinant of hMSC neural differentiation, as also opposite effects were described [23]. The controversy could be explained by the multifactorial triggers needed, apart from 2D vs. 3D spatial contexts, to induce neuronal phenotypes or to maintain or even reverse them to more primitive progenitors. Wharton jelly is a highly mucous tissue that contains high concentration of proteoglycans, mainly substituted with chondroitin/dermatan sulfate chains [24]. High concentration of these chondroitin sulfates for example may be contradictory effector responsible for maintaining cells in their precursor state even in 3D conditions as reported by Canning [25].

Undifferentiated hMSC present in tissue niches in vivo and in the initial WJ fragments in vitro in response to a new but still not well-determined stimulators present in culture environment may undergo spontaneous differentiation [26–30]. In our experiments, it was shown that WJ-MSC cultured in additional presence of hippocampal slices, especially those injured by oxygen-glucose deprivation, significantly accelerate and enhance their spontaneous neural differentiation (Figs. 3 and 4). The critical event seems to be paracrine interaction taking place in this co-culture between hMSC and NSC residing in the rat organotypic culture. NSCs are known to secrete growth factors like BDNF and FGF stimulating WJ-MSC differentiation into the neural direction. Our observation is coherent with Rong’s group [31], where the authors concluded that NSCs promoted BM-MSCs to differentiate into neuronal direction, the most probably via BDNF and NGF stimulatory interaction. Similar interplay was also described by Salgado [32], who underlined the importance of paracrine interactions between hMSCs and NSCs in the stimulated neurogenic niches.

Augmentation of spontaneous neural WJ-MSC differentiation observed here along the increased time of cell passaging could also be partially connected with non-physiological, 21% high oxygen concentration used in these cultures. As described previously by our and other groups [6, 33, 34], atmospheric oxygen conditions accelerate significantly cell differentiation, maturation, and senescence. Other emerging differences between subsequent passages could be associated with the progressive loss of population heterogeneity due to uneven selection of different cell types changing the characteristics of cell interactions and related WJ- MSC secretion.

The changes in the cellular composition during passages in vitro would directly influence the therapeutic properties of the transplantation material [35]. Undoubtedly, the therapeutic efficiency of hMSC transplantation is closely linked with their ability to secrete plethora of different growth and anti-apoptotic factors [36]. WJ-MSCs are well known for their strong trophic activities [37]. Here, we have shown that freshly isolated WJ-MSC secretes relevantly more IL-2, one of the strongest immunomodulatory factors in comparison with cells growing in the fourth passage. Also enhanced here, the secretion of bFGF may lead to further support of the surrounding cell proliferation capacity.

In turn, the WJ-MSC cultures committed toward neural differentiation were reported to secret significantly higher amount of TGFb, IL-10, and VEGF in comparison to freshly isolated cells [38]. It has been shown that combination of IL-6 and TGF-α can also stimulate production of VEGF [39]. These observations confirm the previously described high potential of WJ-MSC in the promotion of vasculogenesis in vitro [38, 40].

The augmented neuroprotective effect of WJ tissue fragments and freshly isolated WJ-MSC (mixed stromal population) over more selected WJ-MSC cultures (p.1–4) correlates with enhanced protective neurotrophic and growth factor gene expression in co-cultured intact or injured rat hippocampal slices. Elevated protein and transcriptional activity of glial cell line-derived neurotrophic factor (GDNF), ciliary neurotrophic factor (CNTF), neurotrophin-3 (NT-3), and hepatocyte growth factor (HGF) were reported to increase the survival of pyramidal neurons in CA1, their axonal outgrowth [41], structural reconstruction [42], and apoptosis prevention. The cell secretive and associated neuroprotective activities would be enhanced too by the coexistence of hMSC and NSC in culture. Haragopal et al. described that co-culture with hMSCs significantly enhance NSC stemness and augment their proliferation rate [43]. The increase described here of the paracrine impact of hMSC-derived vascular endothelial growth factor (VEGF) would have additional protective effect on tissue vasculature. The maintenance of unchanged microvasculature has been shown to be essential for proper function of the neurogenic niches.

In summary, our studies may suggest that WJ-MSC after transplantation to injured brain display substantial differences in their paracrine activities which would be partially dependent from the previous cell culture time and history in vitro. These changes if uncontrolled could be responsible for sometimes awful inconsistency in the results of cellular therapy. The observed here differences in protective properties of the initial WJ tissue fragments and the cells grown along relatively short, four passages of culture period, would be significant enough to have a profound influence on the resulting WJ-MSC therapeutic efficacy and then clinical output after cell transplantations.

## Electronic supplementary material

ESM 1 (GIF 90 kb)

High resolution image (TIFF 105 kb)
