# Supplementary figures and images for: Neuroprotective Potential and Paracrine Activity of Stromal Vs. Culture-Expanded hMSC Derived from Wharton Jelly under Co-Cultured with Hippocampal Organotypic Slices
Source: Mol Neurobiol. 2017 Nov 13;55(7):6021–36. doi: 10.1007/s12035-017-0802-1 (PMC5994221; doi:10.1007/s12035-017-0802-1)

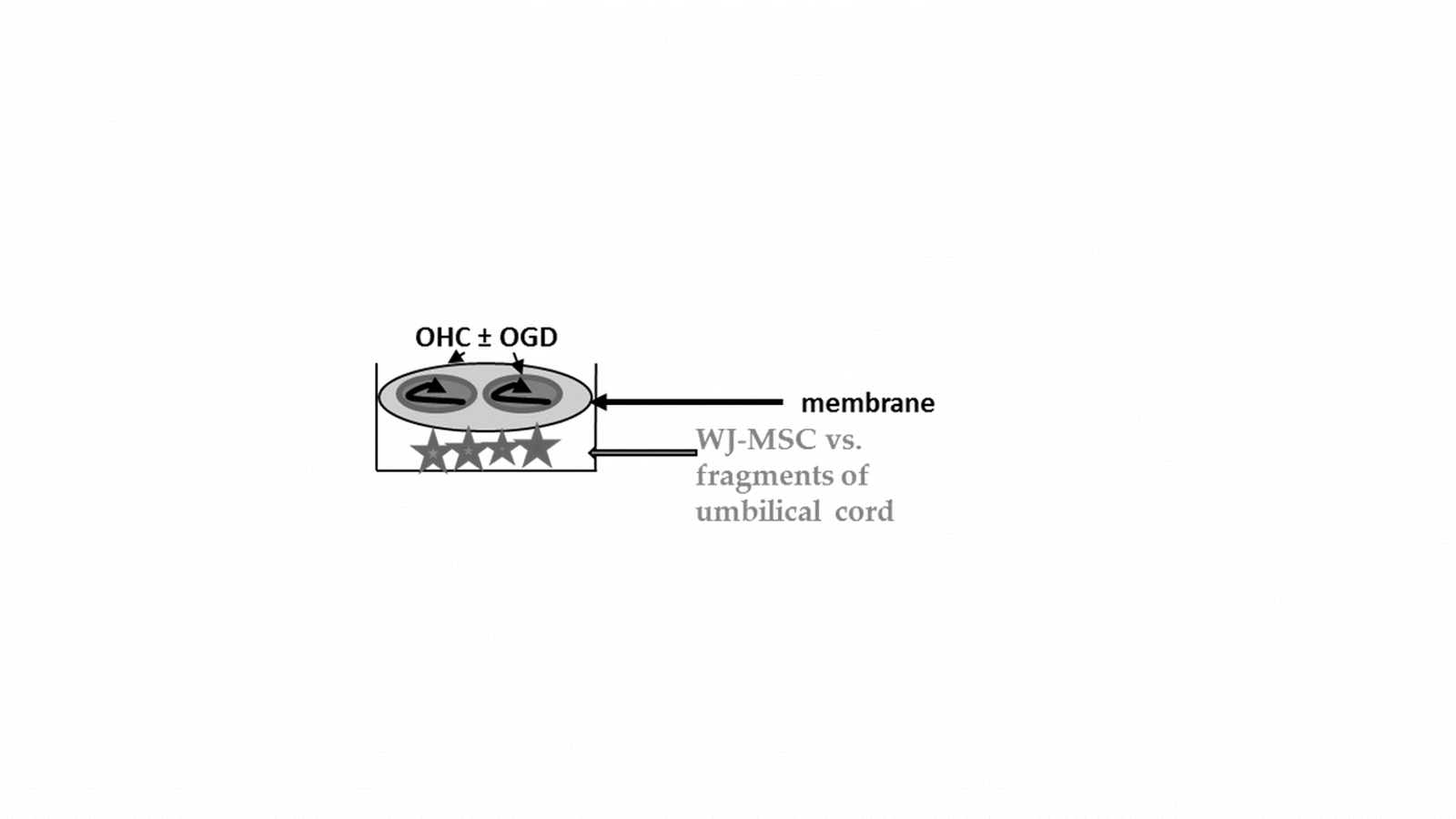

Supplement: Supplementary file 1 — (GIF 90 kb) [file 12035_2017_802_Fig8_ESM.gif]

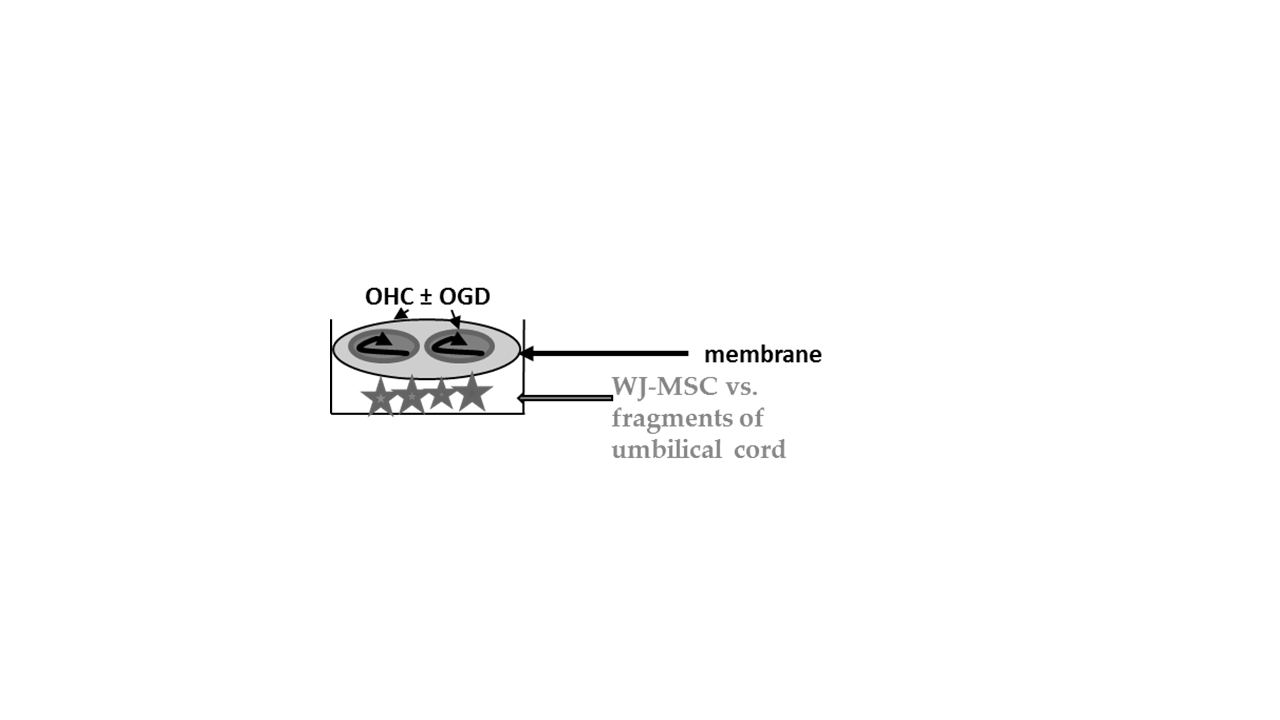

Supplement: Supplementary file 2 — High resolution image (TIFF 105 kb) [file 12035_2017_802_MOESM1_ESM.tif]
